# Assessment of Youth-Friendly Service Quality and Associated Factors in Bole Sub-city, Addis Ababa, Ethiopia: A Cross-Sectional Study

**DOI:** 10.7759/cureus.85042

**Published:** 2025-05-29

**Authors:** Azmera Gebrekidan, Apurvakumar Pandya

**Affiliations:** 1 Parul Institution of Public Health, Parul University, Vadodara, IND; 2 Behavioral Health, Indian Institute of Public Health, Gandhinagar, IND

**Keywords:** awareness, discrimination, output, process, quality, service utilization, stigma, youth-friendly service

## Abstract

Background: Youth-Friendly Service (YFS) quality is a comprehensive YFS standard that encompasses utilization, community participation, teenagers' participation in planning, monitoring, and evaluation, inclusive YFS, and a sense of support and satisfaction with the overall YFS. These output characteristics are instrumental in promoting increased engagement with YFS among youth, communities, and healthcare providers. However, YFS quality is an underexplored dimension within current YFS standards, evidenced by a scarcity of research and limited dissemination of findings, best practices, and policy initiatives. This study was therefore conducted to investigate the quality of YFS and its determinants in Bole Sub-City, Addis Ababa, Ethiopia.

Methodology:* *A total of 365 participants were selected using a multi-stage probability sampling technique. Data were collected through structured questionnaires and analyzed using both descriptive and inferential statistics. Inferential statistics included a one-sample binomial test of proportion and binary logistic regression.

Results: The study findings showed that the YFS quality was sub-optimal, at 46%. Several factors significantly influenced whether YFS quality was rated as good or poor. Specifically, a better YFS process was strongly associated with higher odds of good YFS quality (adjusted odds ratio [AOR] = 1.11, 95% confidence interval [CI] = 1.07-1.14). Increased YFS awareness also led to greater odds of good YFS quality (AOR = 1.03, 95% CI = 1.01-1.04). Conversely, higher levels of stigma and discrimination were linked to lower odds of good YFS quality (AOR = 0.95, 95% CI = 0.93-0.97). Additionally, being female was associated with significantly lower odds of good YFS quality compared to being male (AOR = 0.47, 95% CI = 0.56-3.18).

Conclusions: YFS quality in Bole Sub-City, Addis Ababa, was sub-optimal. This can be attributed to several influencing factors, including gender-related disparities (particularly impacting female beneficiaries), suboptimal YFS process quality, insufficient YFS awareness among beneficiaries, and the presence of stigma and discrimination. These findings critically underscore the importance of not only improving the procedural aspects of YFS delivery but also significantly enhancing youth awareness and actively combating stigma and discrimination within healthcare settings. Therefore, targeted interventions are urgently needed to elevate service standards and ensure equitable, high-quality YFS for all youth.

## Introduction

Addis Ababa is the capital city of Ethiopia with a large, growing number of youth with diverse needs [1]. Youth, particularly in Bole Sub-City of Addis Ababa, face a complex array of sexual and reproductive health challenges, including unprotected premarital sex, early marriage, early pregnancy, unsafe abortions, sexually transmitted infections, drug misuse, and criminal behavior [2,3]. Youth-Friendly Services (YFS) are crucial for addressing these needs, with quality being a paramount requirement for effective service delivery.

Quality of YFS

Quality of YFS is critical for improving utilization of services and increasing the likelihood of obtaining ongoing care [4]. However, existing studies highlight that poor YFS quality emerges from a wide range of factors. These include inadequate and untrained service providers, a lack of privacy and confidentiality, negative attitudes toward providers, and a lack of necessary equipment to provide the essential service package, such as health information, materials, essential drugs, and supplies [4,5]. YFS quality has generally been reported as below average and does not meet the four elements of the World Health Organization’s (WHO) quality standards: equity, appropriateness, acceptability, and accessibility [5].

Different studies across Ethiopia have reported varied statuses of YFS quality. Nationally, for instance, the output quality of Adolescent and Youth Sexual and Reproductive Health Services (AYSRHS) was reported at 57.01% [4]. However, studies from different regions of Ethiopia reveal significant disparities: 47.2% in northeast Ethiopia [6], 33.3% in the Gojam area [7], and 50.50% in Debre Birhan Town [8]. In the Southern Regional State, youth client satisfaction was 49.1% in Arba Minch [9] and 64.9% in the Central Regional States, while East Shewa Zone and Addis Ababa City Administration reported 25.3% YFS satisfaction [5]. This wide variation underscores the importance of context-specific studies like the current investigation, as national averages may mask significant local disparities, necessitating tailored interventions to address regional realities.

For this study, YFS quality is operationally defined as the comprehensive standard encompassing post-service changes observed in the community, among youth, and healthcare facility workers. This includes increased knowledge and utilization of YFS, enhanced community involvement, inclusion of service seekers from diverse backgrounds, active adolescent participation in planning, monitoring, and evaluation, and a collective sense of support and fulfillment derived from the YFS as a whole. While the overall concept of YFS quality comprises input, process, and output standards, the primary outcome variable in this study is derived from the assessment of these post-service 'output' components.

Determinants of YFS

This study investigated specific determinants of YFS quality, grounding its approach within a framework that considered factors influencing service effectiveness. The chosen determinants were YFS process quality, stigma and discrimination, awareness level of YFS users, and gender, each playing a distinct role in shaping the overall quality experienced by youth.

YFS process quality refers to elements of service delivery such as the client-provider relationship, assurance of privacy and confidentiality, and the inclusion of pre- and post-service assessments and reporting. This aspect is considered to have a paramount contribution to YFS quality, as effective processes directly influence client experience and satisfaction. For instance, studies from Ghana [10], Kenya [11], and the United States [12] have consistently shown that improved YFS process quality leads to increased YFS utilization and positive outcomes. These findings emphasize that how services are delivered is crucial for their perceived effectiveness. 

Stigma and discrimination against youth using YFS are significant human rights violations that profoundly impact service utilization and perceived quality [13]. Stigmatization involves the covert exclusion of youth, while discrimination refers to their overt exclusion from various system-oriented life dimensions. This practice negatively affects how teenagers engage with YFS. Evidence from Malaysia [14], Iceland [15], and Sub-Saharan nations [16] illustrates how fear of recognition, breaches of confidentiality, and social exclusion deter youth from accessing essential SRH services. In Ethiopia [17], stigma and discrimination are recognized as major structural barriers to accessing YRHS, directly reducing the perceived quality and effectiveness of services. 

The awareness level of YFS users is another critical determinant, as greater knowledge is strongly linked to increased utilization and satisfaction with adolescent sexual and reproductive health services. Studies from various Sub-Saharan countries, including Ethiopia [11,18], highlight that a lack of awareness is a significant barrier to YFS quality. Conversely, initiatives like multimedia approaches have been shown to increase YFS awareness, leading to enhanced satisfaction and improved YFS quality [19,20]. This suggests that effective dissemination of information about YFS and its benefits is vital for encouraging engagement and positive experiences. 

Finally, gender plays a complex role in influencing YFS quality. Societal expectations and assigned gender roles can directly or indirectly affect the likelihood of youth seeking YFS and their subsequent satisfaction. For example, traditional notions of masculinity may deter men from seeking medical services, particularly those related to SRH, which are often perceived as women's domain [19]. While some studies [5,20,21] indicate that being female is associated with higher YFS utilization and satisfaction, other literature presents mixed findings, with some suggesting a male association with utilization [22]. This complexity necessitates a deeper exploration of how gender influences the perception and experience of YFS quality within specific contexts.

Despite the existing descriptive analyses of YFS quality, a notable research gap exists in understanding the determinants of YFS quality in the study context. Existing literature in Ethiopia has largely focused on service utilization and coverage rather than perceived quality, leaving a gap in understanding service effectiveness from the beneficiary’s perspective. Therefore, the present study aims to (1) assess the level of YFS quality among youth in Bole Sub-City, Addis Ababa, Ethiopia, and (2) identify the determinants of YFS quality among youth in Bole Sub-City, Addis Ababa, Ethiopia. 

## Materials and methods

Study design

This study employed an analytical cross-sectional study design. This design was chosen to investigate the impacts of independent variables, namely YFS awareness, stigma and discrimination, YFS process quality, and gender, on YFS quality.

Study setting

The Bole Sub-City is one of Addis Ababa’s largest sub-cities, ranking fourth in terms of population, and is situated in the city's eastern region. The latest population census in the year 2021 demonstrated that the sub-city has a total population of 425,718, consisting of 198,248 males and 227,470 females [23]. Bole Sub-City is experiencing high population pressure due to rural-to-urban migration [24], and that complicates the youth problems even more than expected. According to the data, the youth population is remarkably high in Bole Sub-City. They are continuously facing an intricate array of multiple challenges, including unemployment [25], gender-based violence [26], and a low level of knowledge and utilization of YFS [27].

Target population

The target population for this study comprised an estimated 1,920 youth residing in Woreda-14 of Bole Sub-City. This population included 820 male and 1,100 female adolescents, distributed across five sub-woredas within Woreda-14 [23]. Table 1 presents the target population of the study.

**Table 1:** Population frame of the study.

No.	Cluster	Male	Female	Total
1	Sub-Woreda-1	162	215	377
2	Sub-Woreda-2	159	216	375
3	Sub-Woreda-3	168	229	397
4	Sub-Woreda-4	168	227	395
5	Sub-Woreda-5	163	213	376
Total		820	1100	1920

Sample size 

To determine the appropriate sample size for the study, the population proportion method was used, assuming maximum variability. The formula for sample size (*n*) under finite population correction is:

 *n* = \frac{*N Z*^2 *P* (1 - *P*)}{*E*^2 (*N* - 1) + *Z*^2 *P* (1 - *P*)}

The proportion (*P*) was assumed to be 0.5. This assumption ensured the greatest sample size and is commonly used in the absence of prior estimates, as it reflects maximum variability. Accordingly, the complementary proportion (*q*) was also set at 0.5. To achieve a 95% confidence level, the *Z*-value used in the calculation was 1.96. The margin of error (*E*) was set at 0.05. Substituting these values into the sample size formula for finite populations, the calculated sample size was approximately 320. Additionally, a 15% non-response rate was considered for this base sample size. This adjustment brought the final required sample size to 368 participants.

Sampling technique

The sample from the total population was drawn through a multistage probability sampling technique. That was a proportional stratified sampling technique followed by cluster sampling, having 50% of variability within the population. The sampling procedure involved several steps. First, proportional stratified sampling was applied to allocate the sample size across the five sub-woredas within Woreda 14 and also by gender within each sub-woreda. The overall proportion used for allocation was approximately 0.1917 (368/1920). Within each stratum, cluster sampling was then employed. Specific clusters (sub-woreda) were randomly selected, and within these selected clusters, gender bifurcation was considered. Individuals (male & female) from each cluster meeting the inclusion criteria were approached. In cases where randomly selected participants could not be reached on the day of the survey, attempts were made to revisit or recontact them. If still unreachable after reasonable attempts, they were considered non-responders. The distribution of the target population and the allocated sample size across sub-woredas with gender bifurcation are detailed in Table 2.

**Table 2:** Sample frame of the study.

No.	Sub-Woreda	Male	Female	Total
1	Sub-Woreda-1	31	41	72
2	Sub-Woreda-2	31	41	72
3	Sub-Woreda-3	32	44	76
4	Sub-Woreda-4	32	44	76
5	Sub-Woreda-5	31	41	72
Total		157	211	368

Inclusion and exclusion criteria

Participants were included if they were between the ages of 15 and 29 years, aligning with the Ethiopian national definition of adolescents and youth. Only individuals who had resided in the selected sub-woredas of Woreda-14 for at least six months before data collection were considered eligible, ensuring that responses reflected familiarity with local health services. Furthermore, participation was limited to those who were willing and able to provide informed consent. For participants below 18 years of age, verbal assent was obtained alongside parental or guardian consent when required. All participants needed to have the capacity to understand and respond to the questionnaire in either Amharic or English, the two languages in which the survey instrument was administered.

Individuals who were critically ill or otherwise incapable of participating effectively in the data collection process at the time of the survey were excluded from the study. This included both physical and psychological impairments that could compromise their communication abilities and participation in the study. Furthermore, youth who were selected through the sampling process but could not be reached after two follow-up visits were not included in the final sample. Lastly, any participants who either declined to participate initially or chose to withdraw their consent during the study were also excluded.

Data collection instruments

The questionnaire served as the primary data collection instrument for the study. Table 3 presents the construct, operational definition, objective of the measure, total items, and response scale.

**Table 3:** Details of the research questionnaire of the study. YFS, Youth-Friendly Service

Sr. no.	Construct	Operational definition	Objective of the measure	No. of items	Response scale
1	YFS Quality: Input	Availability and adequacy of physical infrastructure, materials, and trained personnel in YFS centers.	Assesses availability and adequacy of infrastructure, trained human resources, and relevant materials or resources.	36	1 = Poor (Meaning: Not available or completely inadequate); 2 = Fair (Meaning: Available but with major limitations); 3 = Good (Meaning: Adequately available but with minor gaps);4 = Very Good (Meaning: Available and meets expectations); 5 = Outstanding (Meaning: Fully available, excellent quality).
2	YFS Quality: Process	Experience of service delivery, including interaction quality, confidentiality, waiting time, and responsiveness	To evaluate how services are delivered to youth and their perceived service interaction quality (confidentiality, waiting time, and responsiveness).	24	1 = Poor (Meaning: Extremely unsatisfactory service delivery); 2 = Fair (Meaning: Below average or inconsistent service delivery); 3 = Good (Meaning: Acceptable but with room for improvement); 4 = Very Good (Meaning: High quality and consistent); 5 = Outstanding (Meaning: Excellent and exemplary service).
3	YFS Quality: Output	Perceived effectiveness and satisfaction with the services received at YFS facilities. Effectiveness refers to the self-reported impact of YFS on the sexual & reproductive health, such as increased knowledge, safer behaviors, and improvements in emotional or sexual, or reproductive health.	To determine the level of satisfaction and perceived effectiveness of YFS use.	19	1 = Strongly Disagree (Meaning: I completely disagree with the statement); 2 = Disagree (Meaning: I somewhat disagree with the statement); 3 = Neutral (Meaning: I neither agree nor disagree); 4 = Agree (Meaning: I somewhat agree with the statement); 5 = Strongly Agree (Meaning: I completely agree with the statement).
4	YFS Awareness	Extent of knowledge adolescents and youth have about the availability, scope, and functioning of Youth-Friendly Services.	Measures the respondent’s knowledge and understanding of Youth-Friendly Services availability, its scope, and functioning.	19	1 = Not Aware (Meaning: I do not know this aspect); 2 = Slightly Aware (Meaning: I have heard of it but do not understand it well); 3 = Moderately Aware (Meaning: I have some understanding); 4 = Well Aware (Meaning: I understand this clearly); 5 = Superb Awareness (Meaning: I know very well informed and confident about this).
5	Attitude towards YFS	Perceptions, beliefs, and predispositions of youth towards the usefulness, accessibility, and appropriateness of YFS.	Assesses the participant’s beliefs, feelings, and predispositions regarding the usefulness, accessibility, and appropriateness of YFS.	19	1 = Strongly Disagree (Meaning: I completely disagree with the statement). 2 = Disagree (Meaning: I somewhat disagree with the statement). 3 = Neutral (Meaning: I neither agree nor disagree). 4 = Agree (Meaning: I somewhat agree with the statement). 5 = Strongly Agree (Meaning: I completely agree with the statement).
6	Stigma and Discrimination	The degree to which stigma or discriminatory beliefs are held toward youth using YFS, especially for sexual and reproductive health needs.	Assesses the degree of stigmatizing or discriminatory attitudes toward YFS use.	10	1 = Strongly Disagree (Meaning: I completely disagree with the statement). 2 = Disagree (Meaning: I somewhat disagree with the statement). 3 = Neutral (Meaning: I neither agree nor disagree). 4 = Agree (Meaning: I somewhat agree with the statement). 5 = Strongly Agree (Meaning: I completely agree with the statement).
7	YFS Equity	Perceived fairness and inclusivity of YFS across gender, disability, socioeconomic status, and cultural groups. It focuses on perceived equity in service provision, meaning whether youth believe that all individuals, regardless of their gender identity, disability status, economic background, or cultural affiliation, have equal access to and are treated fairly within the YFS system.	To assess how equitably YFS are provided to diverse adolescent and youth populations.	17	1 = Strongly Disagree (Meaning: I completely disagree with the statement). 2 = Disagree (Meaning: I somewhat disagree with the statement). 3 = Neutral (Meaning: I neither agree nor disagree). 4 = Agree (Meaning: I somewhat agree with the statement). 5 = Strongly Agree (Meaning: I completely agree with the statement).
Total Items	144				

The questionnaire was organized into eight sections, each designed to assess different dimensions relevant to YFS, and was developed based on a comprehensive review of existing literature and validated tools in the field of adolescent and youth health. The first three sections focused on evaluating the quality of YFS through structural (input), process, and output standards. A total of 79 items were adapted from validated sources and distributed across these three domains: 36 items for input, 24 items for process, and 19 items for output. These items were rated on a five-point Likert scale ranging from “poor” (1) to “outstanding” (5), capturing participants' perceptions of infrastructure, provider-client interactions, and service outcomes. The fourth section assessed YFS awareness using 19 self-developed items adapted from prior studies. Participants rated their level of awareness on a five-point scale ranging from “not aware” (1) to “superbly aware” (5). The fifth section measured attitudes toward YFS, consisting of 19 items adapted from previous literature [28]. Participants indicated their level of agreement using a five-point Likert scale from “strongly disagree” (1) to “strongly agree” (5). The sixth section examined stigma and discrimination using 10 self-constructed items, also rated on the same five-point agreement scale, capturing negative perceptions or judgments that may hinder service utilization. The seventh section focused on the perceived equity of YFS, with 17 items developed from multiple sources. These items evaluated fairness and inclusivity across gender, disability status, socioeconomic background, and cultural identity, with responses ranging from “strongly disagree” (1) to “strongly agree” (5). Finally, the eighth section captured sociodemographic information, including seven key domains: age, gender, education, employment status, marital status, childbearing status, and religion.

This comprehensive questionnaire structure, developed based on a thorough review of the literature, enabled a multidimensional assessment of both the perceived quality and accessibility of YFS from the perspective of adolescents and youth. It captured a broad range of constructs, including service inputs, processes, outcomes, awareness, attitudes, stigma, and equity. For illustrative purposes, sample items from each construct are presented to provide a reference for the types of questions included in the tool (Appendix).

Validity and reliability

Experts from public health sciences evaluated the relevance, adequacy, feasibility, and the way each item was phrased, and amendments were made in light of the experts' suggestions. On the other hand, language experts observed the linguistic components of the instruments while there was back-and-forth translation between English and Amharic. Moreover, reliability was tested through a pilot test with a sample of 44 (12%) youth from the total sample size [29]. Based on the findings, items with low internal consistency (*r* = 0.3) were discarded [29]. The sample of the pilot test was not considered for the final sample of the main study.

The questionnaire initially comprised 144 items; however, following validation and reliability testing, the final number of items was reduced to 124. Table 4 presents the constructs, item counts, and corresponding reliability coefficients before and after the pilot test.

**Table 4:** Reliability statistics of the questionnaire. YFS, Youth-Friendly Service

Sr. no.	Variables	Total items before analysis	Cronbach's Alpha before item removal	Total items removed	Total items accepted	Cronbach's Alpha after item removal
1	YFS input quality	36	0.78	8	28	0.87
2	YFS process quality	24	0.90	None	24	0.90
3	YFS output quality	19	0.66	7	12	0.73
4	YFS equity	17	0.80	2	15	0.83
5	Awareness of YFS	19	0.89	None	19	0.89
6	Attitude on YFS	19	0.88	3	16	0.90
7	Stigma/discrimination	10	0.86	None	10	0.86
	Total	144	0.82	20	124	0.85

No changes were made to the number of items in three constructs: awareness, stigma, and discrimination, and YFS process quality, indicating strong initial consistency. For the YFS attitude scale, the original 19 items yielded a Cronbach’s alpha of 0.88; after removing three items, reliability improved to 0.90. Similarly, the YFS utilization scale began with 18 items and an alpha of 0.78; the removal of three items raised the alpha to 0.80. The YFS input quality scale initially had 35 items with an alpha of 0.78; after removing seven items, reliability increased to 0.87. For YFS output quality, the initial 19 items produced an alpha of 0.66, but after discarding seven items, the alpha rose to 0.73. Finally, the YFS equity construct started with 17 items (α = 0.80); the removal of two items improved the reliability to 0.83. Overall, the final tool achieved a Cronbach’s alpha of 0.85, indicating high internal consistency across constructs.

Plan of data analysis

Data were analyzed quantitatively using descriptive and inferential statistics. The descriptive methods particularly focused on frequencies, percentages, and correlation, while the inferential statistics included a one-sample binomial test of proportion and binary logistic regression. The one-sample binomial test of proportion was used to compare a sample proportion to a hypothesized population proportion of YFS output quality and to assess its extent. On the other hand, binary logistic regression was applied to explain and predict the presence or absence of YFS output quality in the study setting.

Ethical considerations

Ethical approval was obtained from Parul University. For participants under the age of 18, assent was obtained along with informed consent from their parents or legal guardians. For those aged 18 and above, informed consent was obtained directly. Strict measures were taken to ensure participant anonymity and confidentiality throughout the study. Data collection procedures safeguarded personal information through the use of anonymized identifiers. Additionally, all participants were informed of their right to withdraw from the study at any time without any negative consequences.

## Results

The study achieved a notably high response rate of 99.2%, with 365 out of the 368 selected participants completing the survey. The minimal non-response rate of 0.8% was attributed to two individuals who could not be reached despite two follow-up visits and one individual who declined participation due to personal reasons.

Demographic information of the participants

This section presents the background information (i.e., demographic variables) of the participants, which mainly includes gender, age, marital status, educational level, and religion. As shown in Table 5, the majority of participants were female (67%), suggesting a higher engagement or willingness to participate in YFS among women. In terms of age distribution, most respondents (71%) fell into the early adulthood category (19-26 years), highlighting that YFS are accessed not only by adolescents but also by young adults. Regarding educational attainment, nearly half (49%) of the participants were at the high school level, with a notable proportion also holding diplomas or degrees (13% each). With respect to employment status, over half of the participants (52%) were students, while 26% were employed, and smaller proportions were either self-employed or unemployed. Marital status data showed that the vast majority (82%) were single. In terms of childbearing, 18% of participants reported having children. Finally, Orthodox Christianity was the most commonly reported religion (61%), followed by Protestantism (24%) and Islam (15%).

**Table 5:** Relevant demographic characteristics of the participants.

Variable	Sub-category	n	%
Gender	Male	119	32.6
	Female	246	67.4
	Total	365	100
Age	Adolescence (15-18 years)	106	29
	Early Adulthood (19-26 years)	259	71
	Total	365	100
Educational Level	Elementary	10	2.7
	Junior	20	5.5
	High School	177	48.5
	Technique	20	5.5
	Diploma	46	12.6
	Degree	46	12.6
	Master	7	1.9
	Total	365	100
Job	Student	189	51.8
	Unemployed	36	9.9
	Self-employed	44	19.1
	Employed	96	26.3
	Total	365	100
Marital Status	Single	299	81.90
	Married	66	18.1
	Total	365	100
Child Bearing	Yes	65	17.8
	No	300	82.2
	Total	365	100
Religion	Orthodox	221	60.5
	Protestant	87	23.8
	Muslim	55	15.1
	Catholic	2	0.5
	Total	365	100

Descriptive statistics and correlation among the main study variables

Table 6 presents measures of central tendency, measures of dispersion, and the relationship between the study variables. A strong, positive, significant relationship was found between input quality and overall quality (*r* = 0.94), overall quality and process quality (*r* = 0.71), and overall quality and output quality (*r* = 0.68). In addition, a moderate positive significant correlation was obtained between input quality and output quality (*r* = 0.51), input quality and process quality (*r* = 0.46), awareness and equity (*r* = 0.46), and attitude and awareness (*r* = 0.40). On the other hand, significant negative correlations were found between stigma/discrimination and input quality (*r* = -0.60), stigma and discrimination and overall quality (*r* = -0.55), and stigma and discrimination and output quality (*r* = -0.43).

**Table 6:** Descriptive statistics and correlation among the major study variables (N = 365).

Variable	Mean	SD	1	2	3	4	5	6	7	8	9
Attitude	51.84	13.17	-	0.40^**^	0.07	0.32^**^	0.04	0.02	0.01	0.03	-0.01
Awareness	66.24	21.52		-	0.10	0.46^**^	0.10	-0.05	0.19^**^	0.10	0.10
Stigma/Discrimination	29.84	15.30			-	0.22^**^	-0.60^**^	-0.17^**^	-0.43^**^	-0.55^**^	-0.51^**^
Equity	44.93	15.08				-	-0.00	-0.08	-0.05	-0.04	0.02
Input quality	61.07	24.40					-	0.46^**^	0.51^**^	0.94^**^	0.58^**^
Process quality	42.30	10.80						-	0.41^**^	0.71^**^	0.32^**^
Output quality	22.58	7.10							-	0.68^**^	0.36^**^
Overall quality	125.94	35.21								-	0.57^**^

YFS output quality

To find out the status of YFS output quality, data were dichotomized into two groups based on the mean value. The mean value of YFS output quality was 22.58. From the minimum score (12) to the lower margin of the mean (22.57), “0” was assigned, referring to no output quality, whereas the scores from the mean (2.58) to the maximum score of the distribution (33) were assigned “1,” denoting that output quality was present. Table 7 shows the present status of YFS output quality. As a result, the data showed no statistically significant difference between the observed and expected proportions of YFS output quality. To put it another way, the present YFS output quality was 46% (95% CI 0.91-0.012, *P* = 0.129).

**Table 7:** One-sample test (N = 365). YFS, Youth-Friendly Service

Variable	Test value = 0.5							
	Mean	SD	*T*	df	Sig. (two-tailed)	Mean difference	95% confidence interval of the difference	
							Lower	Upper
YFS output quality	0.46	0.50	-1.52	364	0.129	0.040	0.091	0.012

Determinants of YFS quality 

Twelve variables were considered to predict the effect on the YFS quality among the participants. Logistic regression was applied to scrutinize the effect of these variables. Table 8 demonstrates that the chi-square model was statistically significant (*P* < 0.001), indicating a significant improvement in fit compared to the null model. This strong model fit suggests that the factors under investigation have a significant effect on YFS output quality.

**Table 8:** Omnibus tests of model coefficients.

		Chi-square	df	Sig.
Step 1	Step	144.68	19	0.000
	Block	144.68	19	0.000
	Model	144.68	19	0.000

The chi-square model was statistically significant (*P* < 0.001), indicating a significant improvement in fit compared to the null model. This strong model fit suggests that the factors under investigation have a significant effect on YFS output quality.

The logistic regression analysis assessed the contribution of each independent variable to the model predicting YFS quality, as indicated by the Wald statistic, significance level, and adjusted odds ratios (AOR). Table 9 depicts findings of logistic regression analysis. Four variables were found to significantly influence perceived YFS quality. First, YFS process quality was positively associated with better service quality perceptions (AOR = 1.108, 95% CI 1.073-1.144, *P* < 0.001), indicating that improvements in service delivery processes significantly enhance perceived quality. Second, YFS awareness emerged as a significant predictor (AOR = 1.030, 95% CI 1.010-1.040, *P* < 0.001), suggesting that higher awareness among youth contributes to more favorable evaluations of service quality. Third, stigma and discrimination were inversely associated with YFS quality (AOR = 0.953, 95% CI 0.932-0.974, *P* < 0.001), indicating that higher levels of perceived stigma lead to poorer service quality perceptions. Finally, gender was also a significant determinant; being female was associated with lower odds of reporting high YFS quality compared to males (AOR = 0.470, 95% CI 0.261-0.848, *P* = 0.012), highlighting potential gender-based disparities in service experiences.

**Table 9:** Variables in the equation. AOR, adjusted odds ratios; CI, confidence interval

Variables	B	S.E.	Wald	df	Sig.	AOR	95% CI for AOR	
							Lower	Upper
Age	0.030	0.066	0.211	1	0.646	1.031	0.905	1.174
Attitude	0.007	0.011	0.409	1	0.522	0.993	0.972	1.015
Awareness	0.027	0.007	12.737	1	0.000	1.027	1.012	1.042
Stigma - Discrimination	-0.048	0.011	17.876	1	0.000	0.953	0.932	0.975
Equity	-0.012	0.010	1.416	1	0.234	0.988	0.968	1.008
YFS Input quality	0.008	0.007	1.147	1	0.284	1.008	0.994	1.022
YFS process quality	0.102	0.016	38.779	1	0.000	1.108	1.073	1.144
Gender (Female)	-0.755	0.301	6.286	1	0.012	0.470	0.261	0.848
Marital status (Single)	0.292	0.440	0.440	1	0.507	1.339	0.565	3.176
Educational status			4.112	6	0.662			
Elementary	-0.756	1.463	0.267	1	0.606	0.470	0.027	8.267
Junior	-0.502	1.327	0.143	1	0.705	0.605	0.045	8.152
Secondary	-1.208	1.178	1.051	1	0.305	0.299	0.030	3.009
Technique	-0.533	1.283	0.172	1	0.678	0.587	0.047	7.261
Diploma	-1.185	1.130	1.101	1	0.294	0.306	0.033	2.799
First Degree	-1.424	1.149	1.535	1	0.215	0.241	0.025	2.290
Job			1.295	3	0.730			
Unemployed	-0.170	0.505	0.113	1	0.737	0.844	0.314	2.271
Student	-0.076	0.409	0.034	1	0.853	0.927	0.416	2.067
Self-employed	-0.625	0.570	1.204	1	0.273	0.535	0.175	1.635
Child bearing (No child)	0.357	0.417	0.730	1	0.393	1.428	0.630	3.236
Constant	-3.758	2.247	2.797	1	0.094	0.023		

## Discussion

YFS quality

This study found that only 46% of youth perceived YFS to be of high quality in Bole Sub-City. Though this was not significantly different from a 50% benchmark, the proportion reflects a suboptimal state of service delivery that warrants programmatic attention. Comparison with national and regional data shows that Bole’s YFS quality is aligned with some regions (e.g., Arba Minch, 49%) [9] but lower than others (e.g., Hosanna Town, 65%) [4]. While the present study reflects a modest improvement over earlier findings from northern Ethiopia [6] and the Gojam area [7], the overall quality of YFS in Bole Sub-City remains below optimal standards and highlights the need for quality enhancement strategies.

Determinants of YFS quality

The first major determinant of YFS quality identified in this study was YFS process quality. The findings affirm that improvements in the quality of service delivery processes, such as maintaining confidentiality, respectful provider-client interactions, adequate counseling, and responsiveness closely associated with higher levels of perceived YFS quality among youth. This positive association is consistent with prior empirical research, including studies from Ghana [10,30] and Southern Ethiopia [31], which highlight that enhanced service processes significantly contribute to better client satisfaction and service effectiveness. These results underscore the need to strengthen the procedural aspects of YFS delivery as a critical step toward improving service quality.

The second significant determinant was YFS awareness, impacting the quality of YFS delivery. Higher levels of awareness about available services and their benefits were associated with increased perceptions of quality. This finding aligns with earlier research, which demonstrated that a lack of sexual and reproductive health knowledge and limited awareness of service availability act as barriers to service uptake and satisfaction [32,33]. Studies conducted in Sub-Saharan Africa and Ethiopia have similarly reported that poor awareness is a key obstacle to YFS utilization [11,18,21]. For instance, evidence from Tigray [18] and Southwest Ethiopia [21] revealed low levels of youth awareness regarding YFS, emphasizing the need for accurate and accessible information. Overall, the data suggest that improved awareness is positively correlated with better service outcomes. Therefore, implementing comprehensive sexuality education, promoting parent-adolescent communication, utilizing social and digital media, training peer educators, and introducing school-based interventions are recommended strategies to enhance awareness and, subsequently, YFS quality.

The third determinant of YFS quality was stigma and discrimination. The findings indicate that higher levels of perceived stigma and discriminatory attitudes negatively influence youth perceptions of service quality. This result is supported by earlier studies from diverse contexts, including Malaysia [14], Iceland [15], and Sub-Saharan Africa [16], which have consistently shown that fear of judgement and social exclusion deters youth from accessing YFS. Studies in Ethiopia have also confirmed that stigma acts as a barrier to service utilization and quality perception [17,33,34,35,36]. Efforts to reduce stigma and discrimination at the community and facility levels are essential for creating a safe, supportive, and youth-friendly environment that encourages service uptake.

The fourth factor was gender influencing YFS quality. In this study, being female was associated with lower odds of perceiving YFS as high-quality, suggesting potential disparities in service experiences across genders. This finding aligns with research suggesting that adolescent girls and young women often face unique structural and sociocultural barriers, such as provider bias, concerns over confidentiality, and stigma related to reproductive health, that negatively influence their service experiences [4,18]. Conversely, some studies have noted that males may be less likely to access or utilize YFS due to prevailing norms of masculinity and a perceived lack of male-targeted services [5,19].

Mixed findings from Ethiopian contexts reflect the variability in gendered experiences of YFS. For example, a systematic review conducted in Ethiopia [20] has reported that female adolescents often experience lower satisfaction due to limited provider responsiveness and privacy concerns. However, other studies from Debre Tabor and Debre Berhan Towns [22,8] and the Amhara region [37] found higher levels of YFS utilization and satisfaction among males. These contradictory findings suggest that gendered experiences of YFS are context-specific and shaped by local service quality, gender norms, and youth mobilization strategies. It calls for gender analysis, and gender-transformative programming is essential for ensuring that YFS are equitable, accessible, and responsive to the diverse needs of male, female, and gender-diverse youth.

Implications for programs

The study offers several implications for enhancing the programming of YFS. The findings of this study underscore the need to strengthen the procedural dimensions of YFS delivery as a foundational step toward improving overall service quality. Key process-related elements, such as respectful provider-client interactions, confidentiality, and timely service, must be prioritized to build trust and enhance young people's satisfaction with health services. Gender-transformative targeted interventions are needed to address the unique needs of all adolescents. Special attention should be given to creating adolescent-friendly spaces that are both physically and psychologically safe, with tailored outreach for marginalized and underserved youth populations, and digital communication tools to increase YFS awareness and utilization [21]. Initiatives that promote open parent-adolescent communication, leverage social and digital media platforms, can significantly enhance adolescents’ knowledge, agency, and access to services. Reducing stigma and discrimination remains central to creating a safe and enabling environment for service utilization. Community-level campaigns, along with provider training focused on empathy and non-discrimination, are critical for ensuring that YFS settings are welcoming and inclusive. Furthermore, the study highlights the importance of addressing gender-specific needs through tailored programming. The gendered experiences of YFS vary by context and are influenced by local norms, service quality, and youth mobilization strategies. Therefore, conducting gender analysis and implementing gender-transformative programming are essential to ensure that YFS are equitable, inclusive, and responsive to the diverse needs of male, female, and gender-diverse youth. 

Implications for policies

The findings of this study emphasize the urgent need for policy reforms aimed at enhancing the accessibility, responsiveness, and equity of YFS, particularly in urban settings like Bole Sub-City, Addis Ababa. Despite ongoing efforts, evidence from Ethiopia and similar contexts consistently shows low levels of reproductive health service utilization among adolescents, often due to systemic, social, and institutional barriers [4,5,38,39,40,41]. To address these gaps, policies must mandate comprehensive adolescent reproductive and sexual health (ARSH) education in both school-based and community settings. This should incorporate culturally sensitive, gender-responsive content that challenges stigma and misinformation, both of which have been shown to hinder service utilization [38,41]. Furthermore, policymakers should prioritize the development and enforcement of standardized YFS protocols that align with the 5A framework proposed by Penchansky and Thomas, ensuring that services are available, accessible, acceptable, affordable, and appropriate [42]. In addition, national and sub-national policies should support capacity-building initiatives for YFS providers. These should emphasize training on adolescent-responsive care, non-judgmental attitudes, and gender sensitivity, particularly for addressing the needs of young women, gender-diverse youth, and vulnerable youth subgroups. Importantly, global evidence warns against uncritical adoption of “best practices” without contextual adaptation. Some interventions, despite being widely endorsed, have proven ineffective, or even counterproductive, when applied without consideration of the local sociocultural landscape [37].

Implications for future research

This study highlights several avenues for future research. First, there is a need for research employing robust methodologies as longitudinal and quasi-experimental designs, to explore the causal pathways linking YFS quality determinants to adolescent health outcomes. Second, future research must center youth voices, particularly those from underrepresented and marginalized groups. Participatory and qualitative methodologies can provide critical insights into the lived experiences of adolescents and the nuanced service delivery gaps they encounter. Such studies should also focus on capturing the dynamic and evolving nature of youth health behaviors, perceptions, and service utilization patterns over time. Third, given the persistent gender disparities in YFS delivery highlighted in this and previous studies [36,38,39,40,41], warrant deeper investigation into gendered service experiences. Research should examine how provider attitudes, structural constraints, and societal expectations affect service use among adolescent girls, boys, and gender-diverse youth. Finally, implementation research is essential to evaluate the real-world effectiveness of policy reforms and programmatic innovations. These studies should assess not only outcomes but also the contextual relevance, scalability, and sustainability of interventions across diverse settings within the local context.

Strengths and limitations

This study possesses several strengths that contribute to its value. It addresses a critical public health the quality of YFS, within a specific and relevant context, the Bole Sub-City in Addis Ababa, where youth face significant health challenges. The use of a quantitative approach with an appropriate sample size and multi-stage random sampling enhances the representativeness of the findings for the study area. The study also utilized standardized scales, and their reliability was rigorously evaluated, with most measures demonstrating high internal consistency.

However, certain limitations should be acknowledged. The cross-sectional design of the study limits the ability to infer causality between the identified determinants and YFS quality; the relationships observed are associative. The reliance on self-reported data introduces potential for social desirability bias, where participants might provide answers they perceive as socially acceptable, and recall bias, which could affect the accuracy of past experiences. The specific geographic focus on Bole Sub-City limits the generalizability of the findings to other regions or national contexts in Ethiopia. Furthermore, while the operationalization of YFS quality was detailed, the dichotomization of the outcome variable using the mean, though a common practice, can lead to some loss of information and may be considered an arbitrary cutoff. Finally, the inclusion of a relatively large number of predictors in the regression model for the given sample size might carry a risk of overfitting, potentially affecting the generalizability of the model to new data. Despite these limitations, the present study offers valuable insights into the local context of YFS delivery in urban Ethiopia and underscores the urgent need for strengthening program implementation, refining research strategies, and informing evidence-based policy formulation.

## Conclusions

This study assessed the perceived quality of YFS and its determinants among adolescents and youth in Bole Sub-City, Addis Ababa. The findings revealed that only 46% of participants rated YFS as high quality, indicating a suboptimal service experience. This result underscores the urgent need to enhance the quality of YFS within urban health systems.

The major determinants for the YFS output quality were the beneficiaries' YFS awareness, stigma and discrimination, YFS process quality, and gender. Higher process quality and greater awareness were positively linked to better service perceptions, emphasizing the value of respectful, confidential, and responsive care. In contrast, stigma, discrimination, and gender disparities - particularly among female participants - emerged as key barriers, reflecting systemic inequities in service delivery. These findings call for targeted interventions to improve procedural dimensions of service delivery, increase adolescent awareness through education and outreach, and address stigma both at the provider and community levels. Furthermore, the gendered nature of YFS experiences points to the need for gender-transformative programming and inclusive policies that recognize the diverse needs of all youth. Overall, the study underscores the importance of context-specific program implementation, evidence-based policy development, and methodologically robust research to advance youth-centered health services.

## Appendices

Appendix:  Example of construct-wise items

YFS Quality-Input

1.   The YFS facility has adequate infrastructure.

2.   The YFS facility has clean and private consultation rooms.

3.   The YFS facility has trained health providers.

4.   YFS providers are trained specifically in adolescent health care.

5.   Educational materials for adolescents are available at the facility.

YFS Quality-Process  

1.   I was greeted politely upon arrival.

2.   The provider did not judge me during the visit.

3.   The provider-maintained confidentiality during the consultation.

4.   I received clear and understandable information.

5.   I did not face long wait times.

YFS Quality -Output

1.   I felt satisfied with the services I received.

2.   My health concerns were fully addressed.

3.   The services met my expectations.

4.   I was able to follow the advice or treatment given by the provider.

5.   I felt more confident about managing my health after the visit.

YFS Awareness  

1.   I am aware that youth-friendly services are available at my local health center.

2.   I know which services are provided under YFS.

3.   I understand the operating hours of YFS facilities.

4.   I am aware of the location of the nearest YFS facility.

5.   I know that YFS includes sexual and reproductive health services.

Attitude Towards YFS

1.   I believe that YFS are essential for improving youth health outcomes.

2.   I feel comfortable seeking services at YFS centers.

3.   I believe YFS staff treat young people with respect.

4.   I believe accessing YFS makes youth more informed about their health.

5.   I believe YFS respects my dignity and rights.

Stigma and Discrimination

1.   I feel seeking sexual and reproductive health services is shameful.

2.   I feel unmarried youth should not access concentrative services.

3.   I feel youth who visit YFS are judged negatively by the community.

4.   I feel talking about sexuality with a provider is inappropriate

5.   I feel visiting YFS damages a young person's reputation.

YFS Equity

1.   The service is equally accessible to boys, girls and gender-diverse youth.

2.   Youth with disabilities are welcomed and accommodated.

3.   Adolescents from all socioeconomic backgrounds feel respected and adequately served.

4.   If culturally marginalized groups or ethnic minorities perceive discrimination in service delivery.

5.   Youth-Friendly Services are sensitive to the needs of diverse gender identities (e.g., youth with Lesbian, Gay, Bisexual or

      transgender identities).
